# Ethics Literacy and “Ethics University”: Two Intertwined Models for Public Involvement and Empowerment in Bioethics

**DOI:** 10.3389/fpubh.2015.00287

**Published:** 2016-02-15

**Authors:** Daniel Strech, Irene Hirschberg, Antje Meyer, Annika Baum, Tobias Hainz, Gerald Neitzke, Gabriele Seidel, Marie-Luise Dierks

**Affiliations:** ^1^Institute of History, Ethics and Philosophy of Medicine, Hannover Medical School, Hannover, Germany; ^2^Institute for Epidemiology, Social Medicine and Health Systems Research, Hannover Medical School, Hannover, Germany; ^3^Health Psychology and Health Education, University of Flensburg, Flensburg, Germany; ^4^Institute of History, Theory and Ethics of Medicine, University of Mainz, Mainz, Germany

**Keywords:** ethics, public involvement, regenerative medicine, public communication, empowerment

## Abstract

**Background:**

Informing lay citizens about complex health-related issues and their related ethical, legal, and social aspects (ELSA) is one important component of democratic health care/research governance. Public information activities may be especially valuable when they are used in multi-staged processes that also include elements of information and deliberation.

**Objectives:**

This paper presents a new model for a public involvement activity on ELSA (Ethics University) and evaluation data for a pilot event.

**Methods:**

The Ethics University is structurally based on the “patient university,” an already established institution in some German medical schools, and the newly developed concept of “ethics literacy.” The concept of “ethics literacy” consists of three levels: information, interaction, and reflection. The pilot project consisted of two series of events (lasting 4 days each).

**Results:**

The thematic focus of the Ethics University pilot was ELSA of regenerative medicine. In this pilot, the concept of “ethics literacy” could be validated as its components were clearly visible in discussions with participants at the end of the event. The participants reacted favorably to the Ethics University by stating that they felt more educated with regard to the ELSA of regenerative medicine and with regard to their own abilities in normative reasoning on this topic.

**Conclusion:**

The Ethics University is an innovative model for public involvement and empowerment activities on ELSA theoretically underpinned by a concept for “ethics literacy.” This model deserves further refinement, testing in other ELSA topics and evaluation in outcome research.

## Background

New developments in biomedical research attract public attention, in particular, biobank-based research and gene transfer methods, as well as biomedical innovations, such as synthetic biology, regenerative medicine, neuroimplants, and nanotechnology. A recent report from the British Nuffield Council on Bioethics associated emerging biotechnologies with a threefold challenge that demands more intensive “public discourse ethics.” The three challenges are (1) “uncertainty” about outcomes; (2) “ambiguity,” meaning disagreement or diverse views and perceptions about the importance, values, and implications attached to biotechnologies; and (3) the “transformative potential” to create large-scale, unexpected changes and disrupt existing technologies, relations, and practices (1).

To address these challenges, leading international institutions stress the importance of public involvement in biomedical research and innovation (2–4). Public involvement activities are often classified into different categories with different approaches and objectives, e.g., information/communication, consultation, and participation/deliberation (3, 5–10).

Public information is usually understood as a *one-way* activity in which scientists provide the public with relevant information on a particular subject “to help them gain knowledge” (8) and ensure that the public can make informed decisions or arrive at an informed opinion. Common methods include websites, information events, and provision of reading material (7). Whereas public consultation is also a one-way activity, although with the information flowing in the opposite direction (from the public to experts), public participation/deliberation is *multi-directional*, inasmuch as it is conceived of as a form of dialog between experts and the public.

Public information is certainly valuable in its own right since it provides lay people with expert knowledge, motivates them to engage in a specific area, and may help them to make better decisions for themselves. However, it can also be valuable as a component of complex, multi-stage public involvement activities that not only consist of informing the public but also of consulting it or even including it in a deliberative event. In a handbook for public involvement in policy making, the OECD acknowledges that information is a “condition for further activities of consultation and active participation to work” [OECD (3): p 52]. Advanced forms of public involvement, therefore, require preparatory information to be delivered to the public in order to be successful. Abelson et al. (9) point out that how participants are informed in a public involvement activity is a crucial criterion for evaluating its success: challenges that need to be addressed in this context include an appropriate degree of accessibility and adequate time for the participants to process the information, but also appropriate decisions regarding *what* information is delivered and *by whom*, in order to avoid potential biases in the process of provision of information (9). Finally, Rowe and Frewer (10) create a link between fairness and efficiency in provision of information: if the information provided to participants in a public involvement activity is biased, this is not only unfair toward them but also compromises the efficiency of the entire activity and is likely to result in a suboptimal outcome. Hence, adequate provision of information is a central challenge for organizers of public information activities, be they organized as isolated events or as parts of more complex public involvement processes.

The purpose of this paper is to introduce a model for a public information event, called an “Ethics University,” that is based on the concept of “ethics literacy” that includes *information* but adds *interaction* and *reflection* as two complementing elements to the idea of competence in ethics. This model combines the concept of “ethics literacy,” which will be presented in detail below, and experiences from the “patient university” (*Patientenuniversität*), an institution at Hannover Medical School, Germany. The patient university was created in 2006 and aims at increasing the knowledge of lay citizens about health-related issues, empowering them to reflect upon these issues in a competent way and to make well-informed decisions. By combining ethics literacy and the patient university, the Ethics University is conceived of as a tool to foster public empowerment in ethical, legal, and social issues in biomedical research and health care.

The model for an Ethics University was piloted in two events in 2012 and 2013 at Hannover Medical School (Germany) and funded by the German Ministry of Education and Research (BMBF). This event invited high school students and apprentices from Lower Saxony, Germany, and focused on regenerative medicine as its topic. The pilot consisted of two series of events, the first conducted in September/October 2012 and the second in January/February 2013, each consisting of 4 days. In addition to the model itself, we will present evaluation data of both pilot events.

Because the public involvement events were not designed as research projects but as deliberative and educative events, the participants do not classify as research subjects and no ethics approval was obtained. Data were gathered via voluntary and anonymous evaluation sheets as used in most teaching or education events.

## The Concepts of “Ethics Literacy” and “Ethics University”

### Concept of “Ethics Literacy”

The concept of “ethics literacy” can be interpreted as a synthesis of the basic ideas of “health literacy” and constituting elements of what Dieter Birnbacher calls “ethics expertise” (11). The concept of “health literacy” is internationally established in public health and health services research (12–14). It basically refers to critical health education or health competence. The internationally accepted definition, supported by the WHO, is: “Health Literacy is the degree to which individuals have the capacity to obtain, process and understand basic health information and services needed to make appropriate health decisions” (12, 13). According to Nutbeam (14), there are three different levels of health literacy that are based on each other: the *functional* level, the *interactive* or *communicative* level and, finally, the *reflexive* or *critical* level.

Similar to health literacy, ethics literacy also needs to develop two further levels that complement the functional level of information about normative theory and factual knowledge, such as relevant information regarding ethical, social or legal challenges of, for example, regenerative medicine. These further levels are necessary in order to develop rational positions and decision-making on ethically complex issues. The levels in question are the levels of *interaction* and *reflection*. The concept of “ethics expertise,” developed by Birnbacher (11), is used as a starting point to make the three levels of ethics literacy more specific. We refer to Birnbacher’s work for two reasons. First, his conceptual work is compatible with the levels of competence in the health literacy concept. Second, Birnbacher’s work has influenced several definitions and analyses on what contributes toward competence in ethics (15–17). Birnbacher distinguishes between the following constituting elements of ethics expertise: (a) information (e.g., ethical theories, principles, concepts, but also knowledge about relevant examples) and (b) specific abilities which can be assigned to the level of interaction. These abilities include (i) empathy for the viewpoints of others and (ii) a tolerance for ambiguities, that is, the ability to endure temporal ambiguities, ambivalence, and hypothetical reasoning. These abilities can be regarded as prerequisites for (iii) the ability of conflict mediation, in which one party is rendered more sensitive toward the viewpoint of another party, so that prejudices and false perceptions can be corrected. Third, Birnbacher refers to (c) certain cognitive standards that can be assigned to the level of reflection. These standards include the individual motivation to adhere to specific cognitive quality standards, such as unambiguity and transparency, as well as logical coherence and consistency.

### Concept of the “Ethics University”

According to the concept of “ethics literacy,” people need to be educated on each of the three levels in order to develop well-informed opinions on ethical issues. The model of an Ethics University aims to educate the participants on each of these levels with respect to a specific bioethical question. Furthermore, the practical relevance of these levels for the formation of ethical judgments must be demonstrated to the participants.

The didactic concept of the Ethics University reflects the idea that learning (a) is a form of functional acquisition of knowledge, (b) is possible as a form of self-perception, and (c) can serve as a correction of internalized patterns of interpretation and value systems (18). It also aims at the acquisition of competences for actions. In order to fuel processes of individual learning, an active involvement – as opposed to passive reception – with the topic of learning is highly important (19). The Ethics University, therefore, combines the principle of structured transfer of knowledge by lectures given by experts with a variety of topic-related “learning stations.” Every topic is introduced by a short lecture and a question-and-answer session, with the content of the lecture then being addressed in greater depth at the respective learning stations. The participants can use the learning stations for gaining knowledge about the topic according to their own preferences, such that the model realizes the principles of participation-oriented didactics. Options offered to the participants include the observation of models, conducting experiments, taking part in tests, and discussions with experts (20). In order to support this process, written or digital materials are delivered to the participants. Researchers from different disciplines, experienced practitioners, and advanced medical students, called “tutors,” serve as assistants and partners for discussions at the learning stations.

### The Tutor Model

The didactic principles of the tutor model are based on two different perspectives: first, “learning through teaching,” which implies that someone learns by helping others in their attempts to learn and by delivering his or her own knowledge to them. This includes the notion that the topic of the Ethics University is closely related to future challenges that the tutors will face in their respective jobs. Second, “learning from peers,” which means that the students can more easily identify with the tutors than with high-ranking experts, for example, because they can communicate with the tutors in their “own language” (21).

Tutors should meet the following requirements: they should be familiar with the content they are expected to teach as well as with the methods that will be used during the activity. Furthermore, they should have role-specific communicative abilities in order to gain the trust of the students. Advanced students of medicine were recruited as tutors for the Ethics University pilot event. They were expected to acquire skills in presenting, explaining, asking questions, and giving qualified feedback. The tutors were prepared for their role in three training sessions, where they were taught presentation and communication techniques and where they were given the opportunity of participating in the exercises that would later be used in the pilot event. The idea was to motivate the tutors to put themselves in the position of being a participant in the pilot event, so that they could anticipate questions and reactions of the actual participants. They were also encouraged to make their own suggestions for improving the project, to assist each other in cases of difficulties, to address questions to the project team, and to discuss potentially difficult situations. See Table/Textbox 1s in Supplementary Material for more detailed information on the content of the tutor training sessions.

### The Topic: Regenerative Medicine

Regenerative medicine aims to repair malfunctioning cells, tissue, and organs by using artificial tissue, on the one hand, and by stimulating regeneration and repair processes, on the other hand (22). It is hoped that this strategy will result in new therapeutic approaches for a broad range of diseases, e.g., neurodegenerative diseases such as dementia, tumor diseases, or metabolic diseases, such as diabetes. Regenerative medicine includes the so-called “tissue engineering,” genetic therapy, and therapeutic cloning. Aspects of regenerative medicine that are the focus of public debates include the clinical relevance of possible innovations, ethical and social challenges of stem cell research and translational research, and the long-term storage of human tissue.

The development of basic, stimulating, but realistic questions was important for the discussions in the Ethics University pilot event. See Table/Textbox 2S in Supplementary Material for more detailed information on the ethical issues of “regenerative medicine.”

### Recruitment of Participants

The participants included high school students as well as apprentices and were recruited through various channels. First, every school, parent speaker, and student speaker in the Hannover region was contacted by e-mail. They were informed about the event and received an Ethics University flyer. The schools themselves were also contacted in a letter sent by post. This contained a cover letter and 20 flyers to be handed to teachers of subjects like religion, biology, and values and standards. In order to further advertise the event, members of the project team also handed out flyers and posters in person at the schools.

A total of 38 schools in Hannover were invited to the first event; 64 schools in the wider Hannover region were invited to the second event. As an incentive, the students were given the opportunity to receive a certificate of participation. A total of 116 students participated in the first event, with 111 students participating in the second event.

## Description of the Ethics University Pilot

The Ethics University consisted of 4 days of 3 h of teaching in the afternoon and early evening. The introductory presentations were addressed to all of the students, the groups at the interactive learning stations and in the group working sessions consisted of 20 students or fewer. These small groups were formed every day in order to foster the discursive process with different people. Every student drew a number at the beginning of each day and was thereby randomly assigned to his or her group. Each of the small groups was accompanied by two tutors.

### Day 1: Introduction and Scientific Basics of Regenerative Medicine

As an introduction to the first day, the students were greeted and invited to document their ideas about “ethics” and “regenerative medicine” by writing key words/notes on posters. This exercise was then repeated on the fourth day to compare their answers and assess them for a possible increase in their knowledge. Subsequently, the project, the schedule for the days, the concept of “ethics literacy,” and the intention behind the project were presented to the students by the project leader.

A presentation about genetic therapy in regenerative medicine by an expert provided the students with basic information about organs, tissues, cells, and cell regeneration. The expert addressed topics like genes, genetic defects, gene therapies, and the idea of gene transfer. He also pointed toward ethical questions with regard to somatic gene therapy. Afterwards, the students were invited to participate in several or all of 10 of the learning stations provided (Table 1).

**Table 1:** **Learning stations for day 1**.

1.	“A view into the body: the heart”: The participants were given the opportunity to learn about the structure and function of an animal heart. They were also able to gain knowledge on the position of the organs in a model of the human body
2.	“A jack of all trades: the liver”: A similar exercise was provided with regard to the liver
3.	“Cells, tissue cells, and organ cells under the microscope”: The participants were given the use of special microscopes to learn about the structure of specific cells (e.g., heart cells or liver cells) and about the structure of muscles or bones
4.	“E-learning module: a view into the cell”: This e-learning module contained information on cells, cell cycles, and stem cells and included short film sequences, drawings, and images. The participants were given the opportunity to use the module on the first day and also received a password for individual studies afterwards.
5.	“The stem cell: what are stem cells?”: An expert gave a short presentation on the properties, differences and similarities of toti-, pluri-, and multipotent stem cells. He supported the presentation with a “puzzle model” of stem cells that involved the participants practically in the presentation.
6.	“A new hope: stem cells” (movie): This short movie presented the aims and projects of the Center for Regenerative Therapies at the Technical University Dresden, Germany, which works on self-regenerative abilities of the human body and aims to develop new, regenerative therapies for as yet incurable diseases
7.	“Degeneration: the deterioration of organs”: The participants were informed about the various causes of arthrosis, a degenerative deterioration of entire joints, including ligaments, bones, and muscles. Models were used to explain regenerative medicine as an approach to cure arthrosis
8.	“Sources of regenerative medicine”: The properties of stem cells and the concepts of toti-, pluri-, and multipotency were briefly explained. Afterwards, the participants learned the difference between embryonic and adult stem cells, e.g., with respect to their developmental potential and their therapeutic usefulness. Furthermore, ethical aspects on the production of embryonic stem cells were presented as well as the basics of induced pluripotent stem cells, the prospects, and the risks of using them for therapeutic purposes
9.	“Regenerative medicine: therapeutic approaches”: On this learning station, an expert gave a brief presentation on stem cell therapy as an alternative to organ transplantation, but also on the risk of the development of cancerous cells. He also addressed ethical issues, like the destruction of embryos in the production of embryonic stem cells and the problem of the beginning of human life.
10.	“The skin factory: a project on wound healing”: The participants learned about a specific project on wound healing that was conducted at Hannover Medical School. They were informed that the webs of a certain species of spider possess properties that contribute to rapid cell growth and could therefore be used in tissue engineering for human nerves

The day concluded with an expert presentation on ethics, with a focus on regenerative medicine. The presentation focused on the difference between ethics and morality and explained ethics as all forms and techniques of discussing moral values and justifications, such as reflection, discourse, and argumentation. Finally, the participants were provided with a handout on ethical principles, values, and relevant questions.

### Day 2: Stem Cells, Embryos, and Associated Ethical Aspects

As an introduction to this day, the participants were asked to illustrate their own opinion on the question “Is it permissible to produce embryos for research purposes?” by indicating their opinion with a sticker on a poster. The poster featured a continuum between “Yes,” “I do not know,” and “No.” This introductory exercise was repeated at the end of the day to see whether (or not) there had been a change in the students’ opinions.

After a review of the first day and a short presentation on the background of stem cell research, the students were assigned to small groups of about 20 and worked on the topic of therapeutic cloning. In this group session, the students were confronted with a fictional scenario featuring a patient suffering from a severe heart disease. The patient is given the option of enduring a long period of time until a transplant is possible or participating in a therapeutic cloning experiment. The students were asked to adopt different roles or perspectives (stem cell researcher, patient, physician, religious wife, and daughter) and to discuss the case in even smaller groups of four to six students. The following questions were discussed: should the patient use the experimental therapy? Is it permissible to produce and destroy an embryo for this purpose? Afterwards, the groups presented their results to each other and discussed this “embryo-destroying” therapy. Finally, the groups discussed their respective results with each other, including the challenges they encountered, and listened to an expert presentation on general ethical aspects of stem cell research.

The second day featured three learning stations (Table 2).

**Table 2:** **Learning stations for day 2**.

1.	“Biobank”: A representative of the Hannover Unified Biobank presented the basic idea of biobanks as well as ethical and legal aspects.
2.	“Informed consent”: This learning station was related to “biobanks” and provided the participants with the opportunity to increase and apply their knowledge. They learned that informed consent is an expression of patient autonomy and a necessary prerequisite for any ethical or legal justification of diagnostic or therapeutic interventions as well as research on humans. Afterwards, the participants were given a fictional consent form and were asked to decide whether they would donate tissue to a biobank and how they would justify their decision.
3.	“Research ethics committees”: The participants were informed about the difference between institutional research ethics committees (RECs) and the clinical ethics consultation. The role of RECs was explained in more detail. Afterwards, participants were asked to adopt the perspective of a member of a REC who has to decide whether testing a new drug that could cure stomach cancer but could also be harmful to the study participants is justifiable. The participants were also asked to consider that benefits and risks have various different aspects and that one should consider the probability of benefits and risks occurring, respectively.
4.	“German Ethics Council”. The background, purpose, procedures, and members of the German Ethics Council were presented.

### Day 3: Allocation of Funding in the German Healthcare System

At the beginning of Day 3, an expert in ethics gave a presentation on ethical aspects of regenerative medicine, focusing on questions regarding humanity, identity, and the body as well as disease and suffering.

The subsequent group session (in 10 groups with 10 participants each) was designed as a simulation of a healthcare conference that had to decide on the allocation of financial funding to projects in regenerative medicine. The participants were asked to allocate funding to research on specific diseases (arthrosis, diabetes, juvenile hair loss, and blindness) from the perspectives of different stakeholder groups. In this healthcare conference, they participated as representatives of health insurance companies, politics, a self-help organization, research, and industrial companies. After presenting the results of discussions among the stakeholder groups, the group as a whole had to decide within a healthcare conference setting on how to allocate the available funding. Afterwards, all groups presented their healthcare conference decisions to each other in a plenary round. To conclude, an expert introduced criteria for the evaluation of research proposals and decision processes in the German healthcare system.

### Day 4: The Future of Regenerative Medicine

At the beginning of Day 4, and similar to Day 1, the participants were again invited to document their ideas about “ethics” and “regenerative medicine” on posters. This exercise was an attempt to enable them to reflect on their answers given on Day 1 and to show how their knowledge had increased over the whole project.

Small groups were formed for discussing the effects of the Ethics University and the idea of ethics literacy. The members of the project team used a structured guideline for moderating the discussions. The discussions complemented the evaluations of each individual day, provided the opportunity to refine the concept of “ethics literacy” and were recorded on tape after obtaining oral consent from the participants.

The Ethics University concluded with an informal get-together among all participants. Parallel to the get-together, tutors recorded short interviews with participants on ethical aspects of regenerative medicine. After obtaining their consent, a short movie consisting of scenes from these interviews was prepared for presentation at a round table discussion on public involvement in biomedical research in May 2013.

In a final presentation, a transplant surgeon and expert in regenerative medicine presented an outlook on the future of regenerative medicine. Thereafter, the participants received their certificates.

## Evaluation

Two hundred twenty-seven participants (22.3% male) with an average age of 20 (range: 16–29 years) participated in the Ethics University. They were recruited from various kinds of schools (*N* = 29) in the Hannover region. We evaluated the process quality and participants’ satisfaction of the Ethics University by voluntary and anonymous evaluation sheets. Discussions with participants at the end of the event that aimed to reflect about perceived improvements in ethics competencies also supported the explorative assessment of the concept validity for “ethics literacy.” Mainly quantitative data on format, content, and structure were collected on each of the 4 days for an evaluation of the event. A complementing evaluation sheet was used for collecting specific quantitative data during the group interviews.

### Evaluation of the Event

The Ethics University was evaluated using day-specific questionnaires. The participants were asked to evaluate the event with respect to the comprehensibility of the presentations and learning stations, the group sessions, and their own success (grade scale from “1” to “6,” with “1” being the best and “6” being the worst possible grade, or Likert scale from “very good” to “very bad”). The main aim of the questionnaires was to find out which didactic formats are particularly suitable for effectively teaching which kind of content. Tables 3–6 present the results of this part of the evaluation.

**Table 3:** **Participants’ ratings of the presentations**.

	Comprehensibility	Structure	Comprehensibility of images and texts	Knowledge gain	Total grade
Grades^a^	2.0	2.0	2.0	2.4	2.1

*^a^The grading system followed the German school grades from 1 for “excellent” to 6 for “insufficient*.”

**Table 4:** **Participants’ ratings of the learning stations**.

	Comprehensibility	Content	Explanations by experts/tutors	Knowledge gain	Total grade
A view into the body: the heart	1.6	1.9	1.7	2.7	2.0
A jack of all trades: the liver	1.4	1.8	1.6	2.3	1.8
Cells, tissue cells, and organ cells under the microscope	2.0	2.2	2.1	2.6	2.2
E-learning module: a view into the cell	1.9	2.4	2.0	2.8	2.3
The stem cell: what are stem cells?	1.7	1.8	2.0	2.8	2.3
A new hope: stem cells (movie)	1.9	2.1	1.9	2.3	2.1
Degeneration: the deterioration of organs	1.7	2.2	2.0	2.7	2.2
Regenerative medicine: therapeutic approaches	1.8	2.0	1.8	2.2	1.9
The skin factory: a project on wound healing	1.5	1.6	1.6	1.8	1.6
Sources of regenerative medicine	1.8	1.8	1.9	1.8	1.8
Biobank	1.8	2.1	2.0	2.0	2.0
Informed consent	1.6	2.9	2.1	2.9	2.4
Research ethics committees	1.9	2.7	2.0	2.3	2.2

**Table 5:** **Participants’ ratings of the group session on therapeutic cloning (Day 2)**.

	Stage I (%)	Stage II (%)	Stage III (%)
Very good	39	38	25
Good	48	48	58
Less than good	12	11	16
Bad	1	2	0
Very bad	0	0	0
N/A	0	1	1

**Table 6:** **Participants’ ratings of the group session on a healthcare conference simulation (Day 3)**.

	Stage I (%)	Stage II (%)	Stage III (%)
Very good	37	21	16
Good	44	47	58
Less than good	15	27	15
Bad	3	5	3
Very bad	1	0	2
N/A	0	0	6

### Small Group Interviews: Evaluation of the Concept of “Ethics Literacy”

The educative and deliberative group interviews on Day 4 aimed to reflect and discuss with all participants the core concept of ethics literacy. By using guided group discussions, the participants expressed and discussed their views on the elements of ethics literacy, which areas of ethics literacy were primarily addressed through the Ethics University, and which aspects they regarded as particularly dominant. The groups consisted of 20 or fewer participants and were moderated by members of the project team. The discussions were recorded on tape after explaining the need to evaluate the performance of the ethics university and the voluntariness for participating in the discussion. All participants gave their oral consent for the recording and anonymized analysis of group discussions.

The participants were asked complementing questions regarding their experience with the 4-day event. First, the participants were asked about their understanding of “ethics literacy”: “What does ‘ethics literacy’ mean to you?” The concept was not explained to them beforehand and the question itself was not previously structured in any way. Afterwards, the participants were confronted with selected results of several working sessions of the Ethics University, which showed that some of them had changed their opinions drastically over the course of the Ethics University.

In the discussions, attitudes and reasons that motivated the participants to make certain decisions were analyzed by the group. The purpose of this part of the discussion was to demonstrate the underlying competences to the participants and to make their own reflections during the Ethics University visible to them.

After a brief repetition of the concept of “ethics literacy” and its three levels – information, interaction, and reflection – the participants were asked to rate these levels with respect to their importance on a scale from “1” (not important at all) to “10” (very important). The information level was rated at 8.31, interaction at 7.56 and reflection at 7.52.

A discussion was also carried out on whether, why, and to what extent they allocated equal or unequal weight to the three levels and whether these should be complemented by further aspects.

Later on, the participants were asked to evaluate how well the Ethics University had educated them on the levels of information, interaction, and reflection with regard to regenerative medicine. They were also asked whether they perceived themselves as more proficient in reasoning about or discussing aspects of regenerative medicine after having participated in the Ethics University. Finally, when, where, and by what means they had gained ethics literacy during the learning sessions was discussed. The knowledge gain was measured based on German high school grades given by the participants, “1” meaning “very good,” “6” meaning “insufficient.” The educational value of the Ethics University with reference to information was rated at 2.2, interaction at 2.6, and reflection at 2.7.

Finally, the participants were asked whether they would be interested in further ethics universities on other topics. Eighty-two percent of the participants responded to this question with “yes” or “tending toward yes.”

Table 7 presents some selected responses from the group interviews.

**Table 7:** **Spectrum of responses in group interviews**.

Question	Keywords	Quotes
What does “ethics literacy” mean to you?	Background knowledge on ethics	“Basic knowledge was important: what plays a role in ethics or which topics are dealt with? What needs to be kept in mind, which aspects play a role?”
	Abilities in reasoning and forming opinions	“The ability to weigh something up and to decide what is right and wrong for oneself”
	Abilities in justification	“First to inform oneself, in order to have well-justified arguments and to ensure that one’s statements are correct”
	Information	“To be informed about a topic and to have formed an opinion”
	Exchange	“The ability to communicate one’s own position and the ability to put oneself into the position of others”
	Reflection	“To reconsider positions again and again, because they change over time – are they still up to date?”
	Contextual knowledge	“Society plays a larger role than one would expect. Religious observations and personal perspectives are often relevant. The Ethics University showed various aspects”
Why do you find these components of ethics literacy more or less important?	Information	“I find this difficult; I think information is most important, because no decision can be made without background knowledge; but overall, everything is important”
	Interaction	“Interaction is most important because everyone already has some ‘preliminary information’. Time for discussion is more important than too much information”
	Reflection	“Reflection is also very important in order to repeat what one has already learned through interaction”
Do you think these three components need to be complemented?	–	“After reflection, I miss a further step – innovation – new formation of opinions – conclusion”
When, where, and by what means did you gain ethics literacy?	Group sessions	“Discussions/role-playing games were helpful (one could get a feeling for weighing something against something else by exchanging with others)”
	Information (presentations, learning stations)	“I learned much from the presentation on therapeutic cloning and stem cell therapy”
	Reflection exercises	“Reflection when placing the colored dots on posters – but elsewhere, one was always able decide for oneself whether one reflected on something or not. In my opinion, there was not very much reflection in the groups, but one did it again for oneself.”
Open questions/feedback	–	“Overall: good; broad information; good insights through unbiased information; how work is conducted”
	Time management	“There was not enough time for those learning stations I approved of. Leave out two stations or restructure them. One could not ask any questions because one would have had to leave the room, but one would still have had to wait outside another room”
	More time for exchange/group sessions	“I expected more freedom for discussions. Furthermore, everything was much too directed towards a particular aim”
	Further topics of interest	“I would participate again, I would be interested in medicine, especially assisted suicide, practical topics, clinical topics”

*The first column shows the question, the second column provides a keyword to the response, and the third column provides direct quotes from the taped recordings*.

With respect to the structure of the Ethics University and the question regarding the participants’ concept of “ethics literacy,” the levels of ethics literacy as developed by the organizers are clearly visible in the responses of the participants. The time management during the Ethics University was criticized and the participants expressed the desire for more opportunities for discussion. On the other hand, the content of the Ethics University as well as the organization of the various components of the event were well received.

## Discussion

The primary aim of the Ethics University to foster the formation of justified opinions in adolescents and young adults was successfully achieved. Evidence for this achievement is found, for example, in statements made by the participants like “Discussions/role-playing games were helpful (one could get a feeling for weighing something against something else by exchanging with others)” or “First to inform oneself, in order to have well-justified arguments and for ensuring that one’s statements are correct” (see Table 7), but also by the fact that 82% of the participants would be willing to participate in a further Ethics University on another topic.

A methodological aim was to improve the model of the patient university that was already established at Hannover Medical School and to combine it with the concept of “ethics literacy” in order to create and to evaluate a new model for public information in the field of ethics. This aim was achieved as well, as evidenced by the favorable comments of the participants. About the same can be said for the concept of “ethics literacy” in general and its appropriateness for providing education in ethical reasoning and argumentation to adolescents and young adults. However, we also acknowledge that there still seems to be room for improvement, as suggested by the comments of various participants.

We believe that two very important aspects of the Ethics University that should be considered for refining are time management and the degree of regulation. Since the informational parts were evaluated as slightly better and slightly more important than the interactive and reflective parts, the latter parts could probably be improved. A first strategy that was suggested by participants is to offer a reduced number of learning stations, so that the participants get the opportunity to work more intensely on specific topics and to have longer conversations with the respective experts and tutors. This strategy would imply a loss of thematic diversity but an increase in depth regarding the remaining learning stations. A second strategy that was also suggested by participants would be to deregulate the group discussions, that is, to give more room for free conversations among the participants. Group moderators would then have to be prepared not to achieve the exercise aims in the discussions, but the participants would have the opportunity to focus on those topics they are personally interested in.

A limitation of the Ethics University was that it exclusively involved high school students and apprentices as participants. This means that the age of the participants was quite low (adolescents and young adults). It cannot be deduced from the evaluation that people who are no longer students or apprentices or who are not adolescents or young adults would benefit from an Ethics University in a similar way, or that they would have similar attitudes toward the concept of “ethics literacy” and the importance of its three components. Further public information activities that apply the model of the Ethics University to participants of other age groups or other school formats are, therefore, a next reasonable research step.

The effectiveness of ethics universities as parts of larger public involvement processes that include consultative or even deliberative components also needs to be assessed empirically. While it is theoretically suggested that public information events like the Ethics University can play a vital role in multi-staged public involvement processes, we suggest that further empirical research is required to determine the value of the model of the Ethics University for such processes.

## Conclusion

This paper presented the model of an Ethics University as a device for public information for adolescents and young adults, with a thematic focus on regenerative medicine. The event itself was generally evaluated positively by the participants, although there remains room for improvement. The same can be said of the concept of “ethics literacy” that the Ethics University was based on: it appears to be valuable for educating adolescents and young adults in ethical reasoning and argumentation about complex issues like regenerative medicine, but it still needs to be assessed with reference to whether it is equally valuable for the education of members of other age groups. The model of the Ethics University, therefore, deserves consideration not only as a valuable instrument for public information but also as an informational component of more complex, multi-staged public involvement processes.

## Ethics Statement

We obtained a retrospective approval of our discourse event and its evaluation from the local research ethics committee at Hannover Medical School on 13th January, 2016.

## Author Contributions

DS and TH wrote the first draft. All authors commented and after revision consented on the final manuscript. DS drafted the idea of the concepts ethics literacy and Ethics University. DS, IH, AM, AB, GH, GS, and M-LD planned, conducted, and analyzed the two pilot events for the Ethics University.

## Conflict of Interest Statement

The authors declare that the research was conducted in the absence of any commercial or financial relationships that could be construed as a potential conflict of interest.

## References

[1] Nuffield Council on Bioethics. Emerging Biotechnologies: Technology, Choice and the Public Good. London: Nuffield Council on Bioethics (2012).

[2] European Commission. Biobanks for Europe. A Challenge for Governance. Brussels: European Union (2012).

[3] Organization for Economic Cooperation and Development. Citizens as Partners. Information, Consultation and Public Participation in Policy-Making. Paris: OECD (2001).

[4] Organization for Economic Cooperation and Development. Planning Guide for Public Engagement and Outreach in Nanotechnology. Paris: OECD (2012).

[5] Involve. Making a Difference: A Guide to Evaluating Public Participation in Central Government. London: Department for Constitutional Affairs (2012).

[6] RAND Europe. Involving the Public in Healthcare Policy. An Update of the Research Evidence and Proposed Evaluation Framework. Cambridge: RAND Corporation (2010).

[7] European Institute for Public Participation. Public Participation in Europe. An International Perspective. Bremen: European Institute for Public Participation (2009).

[8] International Association for Public Participation. IAP2 Spectrum of Public Participation (2007) Louisville: International Association for Public Participation.

[9] Abelson J Forest PG Eyles J Smith P Martin E Gauvin FP. Deliberations about deliberative methods: issues in the design and evaluation of public participation processes. Soc Sci Med (2003) 57:239–51. 10.1016/S0277-9536(02)00343-X 12765705

[10] Rowe G Frewer LJ A typology of public engagement mechanisms. Sci Technol Human Values (2005) 30:251–90. 10.1177/0162243904271724

[11] Birnbacher D Wofür ist der “Ethik-Experte” Experte? In: Gesang B, editor. Angewandte Ethik Aufgaben, Methoden, Selbstverständnis. Paderborn: Mentis (2002). p. 97–114.

[12] IOM. Health Literacy: A Prescription to End Confusion. Washington, DC: Institute of Medicine (IOM) Committee on Health Literacy (2004).

[13] Kickbusch I Health literacy: engaging in a political debate. Int J Public Health (2009) 54:131–2. 10.1007/s00038-009-7073-1 19370309

[14] Nutbeam D. The evolving concept of health literacy. Soc Sci Med (2008) 67:2072–8. 10.1016/j.socscimed.2008.09.050 18952344

[15] Steinkamp NL Gordijn B ten Have HA Debating ethical expertise. Kennedy Inst Ethics J (2008) 18(2):173–92. 18610784 10.1353/ken.0.0010

[16] Schicktanz S Schweda M Wynne B The ethics of ‘public understanding of ethics’ – why and how bioethics expertise should include public and patients’ voices. Med Health Care Philos (2012) 15(2):129–39. 21448745 10.1007/s11019-011-9321-4 PMC3319876

[17] Gesang B Are moral philosophers moral experts? Bioethics (2010) 24(4):153–9. 20394108 10.1111/j.1467-8519.2008.00691.x

[18] Siebert H Didaktisches Handeln in der Erwachsenenbildung. Didaktik aus konstruktivistischer Sicht. Augsburg: Ziel Verlag (2006).

[19] Luchte K Teilnehmerorientierung in der Erwachsenenbildung. Weinheim: Weinheim Beltz (2001).

[20] Dierks M-L Seidel G Angebot und Nachfrage nach kritischer Gesundheitsbildung – Erfahrungen aus der ersten Patientenuniversität in Deutschland. Baden-Baden: Nomos (2009).

[21] Martin JP Lernen durch Lehren: ein modernes Unterrichtskonzept [Internet] (2000). Available from: http://www.lernen-durch-lehren.de/Material/Publikationen/aufsatz2000.pdf

[22] BMBF. Regenerative Medizin und Biologie. Die Heilungsprozesse unseres Körpers verstehen und nutzen. Bonn: BMBF (2005).

