# Supplementary material for: Ethics Literacy and “Ethics University”: Two Intertwined Models for Public Involvement and Empowerment in Bioethics
Source: Front Public Health. 2016 Feb 15;3:287. doi: 10.3389/fpubh.2015.00287 (PMC4753284; doi:10.3389/fpubh.2015.00287)
Supplement: Supplementary file 1 [file Supplementary_Material_1.PDF]

Online supplements

Table/Textbox 1s: Content of the tutor training sessions

During the first training session, organizational issues were discussed. Then the project itself was explained to the tutors before they participated as ‘participants’ in a fictional learning station (see below) that would later be used in the pilot event. The project organizers played the role of ‘tutors’, so that the actual tutors could make suggestions for improving the learning station and to reflect the aims of the pilot event.

In the second training session, the learning stations of the first day of the pilot event were presented to the tutors. They were also assigned to the learning stations in a way that took their personal preferences into consideration. Afterwards, they simulated the group working session that would be a component of the second day of the pilot event. They also received instructions on how to moderate group discussions. The main aspects of this instruction were that the tutors should continuously ask the participants to give reasons for their opinions, that they should not state their opinions, but that they should merely moderate the discussions and reflect the statements of the participants.

The tutors also received an introduction and a handout on ethical principles and basic values as well as relevant questions. Finally, they were prepared to conclude each day with the following questions to the participants:

- What did you notice?
- What was new to you?
- What confused you?
- Which questions are bothering you now?
- What did you miss?

The third training session started with an instruction in communication, especially the method of active listening. The tutors were asked to complete previously prepared statements in an exercise with partners. Their partners listened to them for two minutes, then they repeated the statements in their own words for a further two minutes before switching roles. Afterwards, the experience of being a speaker and a listener, respectively, were discussed among the whole group. The tutors were also given a presentation on how to successfully prepare a moderation, how to react to questions and how to communicate successfully. The group working session of the third day of the pilot event was simulated jointly with tutors. At the end of this training session, the fourth day of the ethics university was presented by the project team.
