# Supplementary material for: Ethics Literacy and “Ethics University”: Two Intertwined Models for Public Involvement and Empowerment in Bioethics
Source: Front Public Health. 2016 Feb 15;3:287. doi: 10.3389/fpubh.2015.00287 (PMC4753284; doi:10.3389/fpubh.2015.00287)
Supplement: Supplementary file 2 [file Supplementary_Material_2.PDF]

Table/Textbox 2s: Ethical, legal and social issues in regenerative medicine

Relevant international literature on this mostly consists of review articles on the scientific basics and on the related ethical issues. The concept of 'regenerative medicine' [20] is regarded as a new paradigm [21] that arose from basic research on therapeutic cloning, genetic therapy, stem cell research, and tissue engineering. Some scientific review articles even include sections that explicitly address ethical issues [22], usually discussing aspects fairly close to research, such as market access, patents, and the role of basic research in general. These issues include insights regarding limits and unsolved problems of techniques that are currently in development. One area in research ethics refers to controversial questions in the context of biobanking [23-25] and translational research [26-28]. Other articles regard these developments as forms of fragmentations of our concept of humanity. This contributes towards the trend of replacing the idea of holistic healing with the idea of repairing, both in the self-perception of medicine as a profession and in the general public. The concept of 'repairing' implies a search for 'spare parts', so a clone that was 'manufactured' for therapeutic purposes could be regarded as a source of such spare parts. Various review articles reflect these or similar perceptions from an ethical perspective [29, 30]. This includes questions on social and political mechanisms for regulation and governance with respect to emerging technologies in the area of regenerative medicine [31-33].
